# Risk stratified treatment for childhood acute lymphoblastic leukaemia: a multicentre observational study from India

**DOI:** 10.1016/j.lansea.2025.100593

**Published:** 2025-05-13

**Authors:** Manash Pratim Gogoi, Parag Das, Nandana Das, Soumyadeep Das, Gaurav Narula, Amita Trehan, Sameer Bakhshi, Venkatraman Radhakrishnan, Rachna Seth, Prashant Tembhare, Man Updesh Singh Sachdeva, Anita Chopra, Shirley Sundersingh, Mayur Parihar, Rahul Bhattacharya, Shripad Banavali, Vaskar Saha, Shekhar Krishnan

**Affiliations:** a Clinical Research Unit, Tata Translational Cancer Research Centre, Tata Medical Center, Kolkata, West Bengal, 700160, India; b Department of Statistics, Bidhannagar College, Kolkata, 700064, India; c Department of Pediatric Oncology, Tata Memorial Centre, Tata Memorial Hospital, Mumbai, Maharashtra, 400012, India; d Homi Bhabha National Institute, Mumbai, Maharashtra, 40094, India; e Pediatric Hematology-Oncology Unit, Department of Pediatrics, Advanced Pediatrics Center, Postgraduate Institute of Medical Education and Research, Chandigarh, 160012, India; f Department of Medical Oncology, BR Ambedkar Institute Rotary Cancer Hospital, All India Institute of Medical Sciences, New Delhi, 110029, India; g Department of Medical Oncology, Cancer Institute (WIA), Adyar, Chennai, Tamil Nadu, 600020, India; h Department of Pediatrics, All India Institute of Medical Sciences, New Delhi, 110029, India; i Hematopathology Laboratory, ACTREC, Tata Memorial Centre, Homi Bhabha National Institute, Mumbai, Maharashtra, 410210, India; j Department of Hematology, Postgraduate Institute of Medical Education and Research, Chandigarh, 160012, India; k Laboratory Oncology, All India Institute of Medical Sciences, New Delhi, 110029, India; l Department of Oncopathology, Cancer Institute (WIA), Adyar, Chennai, Tamil Nadu, 600020, India; m Cytogenetics Department, Tata Medical Center Kolkata, West Bengal, 700160, India; n Department of Statistics, University of Calcutta, Kolkata, 700019, India; o Department of Paediatric Haematology and Oncology, Tata Medical Center, Kolkata, West Bengal, 700160, India; p Division of Cancer Sciences, School of Medical Sciences, Faculty of Biology, Medicine and Health, University of Manchester, Manchester, M20 4GJ, UK

**Keywords:** Acute lymphoblastic leukemia, Paediatric, Treatment, Low middle income countries

## Abstract

**Background:**

Overall survival rates of children with acute lymphoblastic leukaemia (ALL) in high-income countries approach 90%. Treated on the same protocols, outcomes in India, were ∼65%.

**Methods:**

The Indian Childhood Collaborative Leukaemia (ICiCLe) group used genetics and measurable residual disease (MRD) to categorise B-cell precursor (BCP) ALL as standard (SR), intermediate (IR) and high-risk (HR) to receive increasing intensity of therapy. T-ALL were treated uniformly. Data on risk stratification, deaths and relapses were collected annually.

**Findings:**

2695 patients aged 1–18 years were enrolled between January 2013 and May 2018. Induction deaths were significantly lower in SR patients (p = 0·002) compared to others. At a median 61 (59–62) months, the 4-year event free and overall survival was 76% (72–79%) and 88% (85–90%) in SR; 70% (66–74%) and 80% (77–83%) in IR; 61% (51–64%) and 73% (70–76%) in HR; and 69% (62–75%) and 77% (70–83%) in T-ALL patients (p < 0·0001). For BCP-ALL, regression analyses showed age, white cell count, bulky disease, high risk genetics and treating centre as independent prognostic variables. The cumulative incidence of treatment deaths (TRD) and relapses at centres varied from 2% (1–5) to 13% (10–17) (p ≤ 0·0001); and 21% (17–26) to 45% (39–51) (p ≤ 0·0001) respectively with significant differences in proportion of BCP-ALL patients with MRD ≥ 0·01% (p = 0·0007) and time to relapse (p = 0·0001).

**Interpretation:**

Risk stratified directed reduced intensity treatment and collaboration decreases treatment deaths and relapses. Standardisation of genetic and MRD tests across centres and access to high quality drugs will lead to further improvements in survival.

**Funding:**

DBT-Wellcome; 10.13039/501100000732UKIERI, TCS Foundation.

**Research in context**

**Evidence before this study**

We searched for articles published in PubMed before 2013 for outcomes of children with acute lymphoblastic leukaemia (ALL) in India. The search was further refined to only include studies published in the preceding decade where data was available on at least 200 children serially treated with the same protocol and where survival rates for the whole cohort was reported. Five studies were identified. Three studies reported outcomes on the non-risk stratified MCP841 protocol. Of these, one reported on the experience of three centres, with toxic deaths of 11%–23% and event free survival (EFS) of 41%–60%. Two single centre studies also using the MCP841 protocol reported treatment related deaths (TRD) of 24% and EFS of 52%–70%, though median duration of follow up was not available. One centre used the United Kingdom ALL 2003 protocol, subsequently modified to decrease toxicity. In this modification, all BCP-ALL patients received anthracycline free induction with only those with a M3 marrow at day 15 receiving daunorubicin and the second delayed intensification block was withdrawn. With this strategy, TRD lowered from 38% to 21% and EFS improved from 51% to 66%. One centre used a modified Berlin Frankfurt Münster 95 protocol. They did not report on TRD but observed an EFS of 63% at a median follow up of 33 months. None of these were conducted as a formal prospective study with data analysed retrospectively. These studies spanning 1990 to 2011 collectively reported on the outcomes of 1958 children with ALL (Supplementary Table S11).

**Added value of this study**

This is the first collaborative clinical study in children with ALL in India using genetic and measurable residual disease risk stratification to decrease the intensity of treatment in low-risk ALL and standardising treatment across all centres. Data was collected prospectively with a pre-tested form. With centres located across India, it was possible to reassure parents that their children were receiving standard of care and facilitated relocation back to their closest centre. The group, with no formal clinical trial structure or funding, were able to demonstrate high level of concordance to protocol adherence, risk stratification and collecting high quality data. Centres found the resources to successfully introduce fluorescence *in situ* hybridisation tests for genetic analyses and flow cytometry for DNA ploidy and measurable residual disease (MRD). With a systematically treated cohort across centres, innovations at individual centre were quickly adopted across the group. Co-operatively the group overcame regulatory hurdles, convinced review boards and national supervisory bodies to approve the study, and instituted principles of good clinical practice for clinical trials at all centres. Collectively the experience acquired allowed the subsequent launch of a funded randomised controlled trial, the first of its kind in India.

**Implications of all the available evidence**

This study demonstrates that the first step in improving outcomes of children with ALL in low resource settings is to decrease treatment related deaths. This can be achieved by establishing a common national treatment protocol, so all patients benefit equally. Induction deaths in children aged <10 years receiving daunorubicin in induction were double that of those receiving an anthracycline-free induction. Risk stratification allowed de-escalation of therapy in low-risk patients decreasing toxicity. Combined, this strategy engaged non-government organisation support and advocacy for patients contributing to low abandonment rates. Children identified as standard risk and receiving lower intensity treatment had EFS and overall survival of 76% (72–79%) and 88% (85–90%), significantly (p ≤ 0·0001) better than those treated with more intensive therapy. Nevertheless, relapse rates were higher than expected. To improve outcomes further, reasons for the differences in TRD and relapse between centres require further investigation. Possible causes which are remediable include standardisation of genetic and MRD testing, availability of high-quality drugs and optimising maintenance therapy.

## Introduction

Outcomes of children with acute lymphoblastic leukaemia (ALL) treated with modern risk-stratified treatment combination chemotherapy in high income countries (HIC) approach 90%. In India, between 1984 and 1997, the Cancer Institute (CI), Chennai, Tata Memorial Centre, Mumbai (TMC-M) and All India Institute of Medical Sciences, Rotary Hospital, New Delhi (AIIMS-A), shared a common protocol developed in collaboration with the National Cancer Institute, MCP-841. Subsequently centres in India, adapted protocols from the United Kingdom (UK)^1^^,^^2^ or Berlin Frankfurt Münster (BFM) groups.^3^^,^^4^ While survival improved and treatment related deaths (TRD) decreased from the 1970's, outcomes remained at ∼65% over the last 3 decades at India's premier paediatric oncology centres.^1^, ^2^, ^3^, ^4^, ^5^, ^6^ Reported TRD's were 15–30%, with a third or more of these occurring in induction, with relapse rates of 22–41%. Indian centres reporting less-than-optimal outcomes were tertiary level facilities with extensive clinical and diagnostic expertise. Financial support for patients were widely available through governmental and non-governmental organisations (NGO). NGOs provided advocacy, out-of-pocket expenses, and free accommodation to families travelling to specialised centres, leading to low treatment abandonment rates. Most drugs for childhood ALL are manufactured in India and are easily available. Thus, the reasons for the continuing inferior outcomes were not immediately obvious.

In 2012, 6 centres TMC-M, CI, AIIMS-A, AIIMS Department of Pediatrics (AIIMS-B), Postgraduate Institute of Medical Institute of Medical Education and Research (PGIMER), Chandigarh and the Tata Medical Center, Kolkata (TMC-K) formed the Indian Childhood Collaborative Leukaemia (ICiCLe) group. These advanced paediatric oncology centres are located geographically in north (AIIMS-A/B and PGIMER), west (TMC-M), east (TMC-K) and south (WIA) India. From 2013 to 2018, the centres worked together to establish a genetic and measurable residual disease (MRD) risk-stratified approach to treat childhood ALL in India. At the time, Drugs and Cosmetics Act India (1946) required full compensation for clinical trials. Thus the first study reported here was an observational study. In 2016 with the Act amended to exclude academic clinical trials alongside funding from the Indian Council of Medical Research and the National Cancer Grid, a formal randomised open label-controlled trial, ICiCLe ALL-14/InPOG-ALL-15 (CTRI/2015/12/006434) opened to phased recruitment at the different centres.^7^ Here, we report findings from an observational cohort study conducted prior to the start of the formal trial.

## Methods

### Study design and participants

Patients 1–18 years with newly diagnosed ALL were eligible for treatment on the study protocol. All six centres participated in the study (Centres 1–6). Diagnostic laboratory studies included bone marrow tests, cerebrospinal fluid (CSF) studies, leukaemia cytogenetics and assessments of treatment response (Supplementary Table S1A). Patients with Down syndrome, mature B-cell and ambiguous lineage leukaemias were excluded. Patients with T-ALL (including T-lymphoblastic lymphoma) were treated with high-intensity chemotherapy and not risk-stratified. Patients with B cell-precursor acute lymphoblastic leukaemia (BCP-ALL) were stratified as standard- (SR), intermediate- (IR) and high-risk (HR).^7^

Risk stratification variables in BCP-ALL patients included National Cancer Institute risk (NCI standard and high risk), disease bulk (based on lymph node and liver/spleen sizes), central nervous system disease (CNS3 status), high-risk cytogenetics (*BCR*::*ABL1*, other ABL-class fusions, *KMT2A* rearrangements, hypodiploidy, iAMP21, *TCF3*::*HLF1*), prednisolone response at treatment Day 8 (good or poor), remission status at end of induction (treatment Day 35) and levels of bone marrow MRD at end of induction (EoI). Stratification included provisional risk stratification at Day 8 and final risk stratification at Day 35 (following the induction treatment phase) (Supplementary Table S1B).

HR patients included BCP-ALL patients with high-risk presentation features (CNS disease, high-risk cytogenetics) and/or unsatisfactory treatment response (poor prednisolone response at Day 8 (PPR) and SR/IR patients with MRD^hi^ (≥0·01%) at EoI. Provisional SR included BCP-ALL patients with NCI standard risk and non-bulky disease, no high-risk presentation features and good prednisolone response (GPR). Provisional IR included BCP-ALL patients with NCI high risk and/or bulky disease, GPR and no high-risk presentation features. Provisional SR and IR patients with EoI-MRD^hi^ were stratified as HR and treated as such post-induction.

Treatment consisted of intensive treatment phases (induction including a 7-day prednisolone prophase, consolidation, interim maintenance, delayed intensification) followed by a 96-week maintenance treatment phase (Supplementary Table S1C). Treatment intensity was based on risk and varied among the risk groups during the first three treatment phases. Delayed intensification and maintenance treatment phases were common for all patients. Higher treatment intensity included anthracycline treatment and additional asparaginase doses in induction; cyclophosphamide and cytarabine treatment in consolidation (along with additional vincristine and asparaginase doses as part of augmented consolidation) and intravenous methotrexate in interim maintenance (as escalating schedule without leucovorin rescue or as high doses with rescue). Intrathecal methotrexate was administered in all phases of treatment and intensified in patients with CNS disease. Cranial radiotherapy was restricted to patients with CNS disease. Patients with *BCR::ABL1* and other ABL-class fusions additionally received an ABL tyrosine kinase inhibitor.

### Procedures

Diagnostic bone marrow studies included cytomorphology, flow cytometry and cytogenetic studies. Cytogenetic tests evolved over the course of the study and included fluorescence *in situ* hybridisation (FISH) for diagnosis of sentinel gene fusions, gene rearrangements, and iAMP21; DNA ploidy analysis by flow cytometry for diagnosis of aneuploidies and where available, karyotyping (GTG-banding). At some centres, especially earlier in the study, reverse transcription PCR was used to detect leukaemia fusion transcripts. Diagnostic CSF examination was performed at treatment Day 8 and involved haemocytometer cell count and cytospin microscopy. Treatment response at day 8 was evaluated by microscopy enumeration of circulating blasts following 7 days of prednisolone monotherapy (60 mg/m^2^/day). Bone marrow MRD levels were estimated using multiparameter flow cytometry, initially for BCP-ALL (8–10 antigen panel) and later for T-ALL.

### Outcomes

Data was collected annually by the coordinating centre using predefined datasets, collated and verified for accuracy and completeness. Data inconsistencies and missing information were followed up with the relevant centres. Final checks were completed in July 2022. The primary objective was to assess whether risk stratification improved survival and decreased treatment related deaths in low-risk patients. The secondary objective was to examine the variations in events and outcomes among centres. Events included induction failure (non-remission), TRD and relapse. Patients with induction failure had bone marrow blast count post-induction ≥5% blasts and/or other non-remission features. TRD included deaths in induction and post-induction and was the only measure of treatment-related toxicity. Treatment abandonment was defined as treatment discontinuation for ≥3 months. Loss to follow-up was defined as no update on post-treatment remission status for >12 months and these patients were censored at last follow-up. Event-free (EFS) and overall survival (OS) were estimated from end of induction for BCP-ALL risk groups and from time of diagnosis for other analyses. Relapse and TRD were considered competing risks in cumulative incidence estimations.

### Statistical analyses

Descriptive statistics included proportions, median values (with interquartile range), and point estimates with confidence intervals. Group comparisons were made using the Kruskal–Wallis (for continuous data) and Pearson chi-square (for categorical data) tests. Survival analyses employed the Kaplan–Meier method and groups were compared using the log-rank test. Median follow-up duration was determined from the Kaplan–Meier estimate of time to censoring. Cox multivariable regression was used to identify independent predictors of survival. Competing risk modelling was used to analyse relapse and TRD, with the Gray test used for comparing groups and the Fine–Gray test to identify independent covariates. Statistical analyses were performed using R (version 4·4·1), SPSS (version 25), and Stata (Basic Edition 18) software packages, with visualisation performed using R and GraphPad Prism (version 8). Details of R-based analyses are available in Supplementary Information.

This study received ethical approvals from Institutional Review Boards at each participating center. In the case of the study coordinating centre (Tata Medical Center), approval was provided by the Tata Medical Center-Institutional Review Board on October 28th, 2013 (reference EC/TMC/12/13). Informed consent was obtained from all study participants prior to start of treatment.

### Role of the funding source

The study's funding sources were not involved in the research design, data collection, analysis, interpretation, or writing of the report.

## Results

**Fig. 1:**
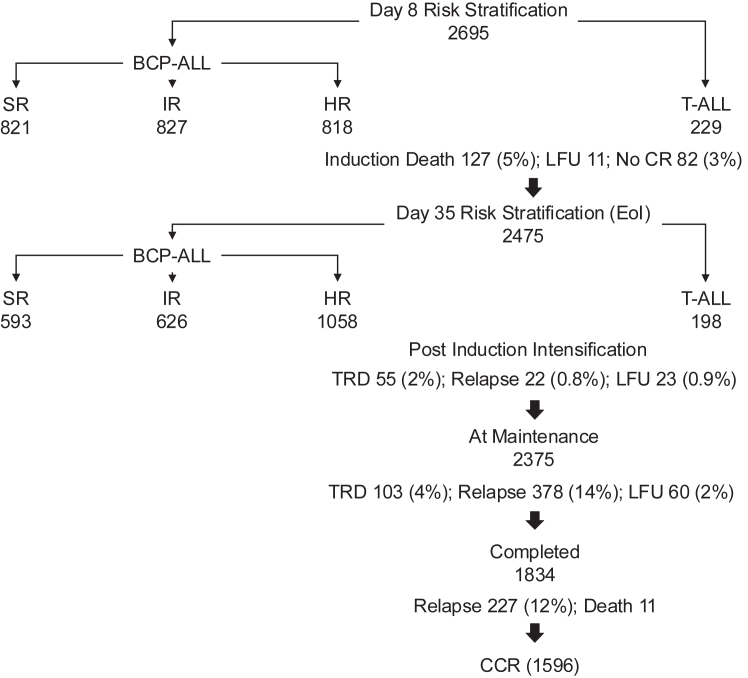
Schematic of numbers of patients with acute lymphoblastic leukaemia at day 8 and 35 of induction and subsequent events pre- and post-maintenance therapy. Eleven patients were lost to follow-up (LFU) in induction, including 7 who abandoned treatment and 4 who moved to other hospitals. Eleven patients died following completion of treatment; while these patients were recorded to be in continuous clinical remission (CCR), the reasons for death were unknown. BCP-ALL, B cell-precursor acute lymphoblastic leukaemia (BCP-ALL); SR, IR and HR, standard, intermediate and high risk respectively; CR, complete remission; TRD, treatment related deaths.

Between January 2013 and May 2018, 2695 patients were treated on the ICiCLe-ALL multicentre protocol. Centre 1 did not enrol T-ALL patients. The median age was 5-years (IQR, 3–9), with a ∼2-fold higher proportion of boys (boys:girls, overall, 1·93, range: 1·63–2·83).^8^ CNS disease was diagnosed in 87 (3·3%)/2594 patients (inter-centre range, 1–4%). High risk cytogenetics was reported in 271 (11·5%)/2364 BCP-ALL patients (range, 5–16%). Poor prednisolone response (PPR) was observed in a sixth of BCP-ALL patients (400/2367, 17%; range 10–36%). Provisionally on treatment Day 8, approximately a third of BCP-ALL patients were each categorised as SR (821/2466, 33%), IR (827/2466, 34%) and HR (818/2466, 33%) (Fig. 1). 2458 (97%; range, 92–99%) of 2540 (94%) patients assessed, achieved complete remission (CR1) at EoI. EoI-MRD^hi^ was reported in 497 (23%)/2166 of BCP-ALL patients. Proportion of EoI-MRD^hi^ varied significantly between centres (11–30%, p = 0·0007). Post-induction, final risk stratification categorised nearly half of BCP-ALL patients as HR (1058/2277, 46%) with the rest divided between final SR (593, 26%) and final IR (626, 28%) (Table 1). Information required for provisional risk stratification was incomplete in 296 (12%)/2466 BCP-ALL patients (Supplementary Table S2). These patients were provisionally stratified according to other available data rather than automatically classified as HR. In patients with complete information for provisional risk stratification, risk allocation was discordant in 35 (1·6%)/2170, with the majority (24/35, 69%) categorised as HR based on physician decision. EoI-MRD levels were available for 1493 (96%)/1557 provisional non-high risk BCP-ALL patients; in the 64 patients with unavailable EoI-MRD, only 7 were treated subsequently as HR (Supplementary Table S3). Treatment abandonment rates were low (3%, 90/2695 patients) and missing information for each of the key risk variables, <5% (Supplementary Table S4).

**Table 1:** Patient characteristics, risk stratification, treatment response and events, overall and by treatment centre.

Characteristics	All centres	Centre 1	Centre 2	Centre 3	Centre 4	Centre 5	Centre 6	p value
**Age in years**	2695	1393	209	342	364	274	113	<0·0001
Median (IQR)	5 (3–9)	5 (3–8)	4·9 (3–10)	7 (4–13)	4 (3–8)	7·5 (3–13)	5 (3–9)	
**Sex**	2695	1393	209	342	364	274	113	0·0088
Male: Female	1·93	1·8	1·71	2·14	2·83	1·63	2·05	
**T-ALL (%)**	229	–	30 (13)	67 (20)	52 (14)	57 (21)	23 (20)	0·1015
**WBC × 1**0^9^**/L**	2692	1393	207	342	364	274	112	<0·0001
Median	12·4	9·3	15·2	17·7	20	15·5	10·6	
IQR	4·6–47	4·2–34·1	7·6–42	4·9–70·1	6–74·8	4·9–66	4·4–56·3	
**CNS disease**	2594	1347	202	302	364	269	110	0·1028
Yes (%)	87 (3)	57 (4)	5 (2)	10 (3)	5 (1)	6 (2)	4 (4)	
No	2507	1290	197	292	359	263	106	
**Cytogenetics, (BCP-ALL)**	2364	1386	175	232	279	209	83	<0·0001
High Risk (%)	271 (11)	191 (14)	9 (5)	37 (16)	13 (5)	12 (6)	9 (11)	
**Prednisolone poor response**	2575	1326	183	335	351	269	111	0·0004
Overall (%)	446 (17)	207 (16)	22 (12)	65 (19)	67 (19)	48 (18)	37 (33)	
BCP-ALL (N)	2367	1326	165	270	304	214	88	
PPR B Cell (%)	400 (17)	207 (16)	17 (10)	48 (18)	54 (18)	42 (20)	32 (36)	<0·0001
**Day 8 risk stratification**	2466	1393	179	275	312	217	90	<0·.0001
BCP-ALL Standard Risk (%)	821 (33)	426 (31)	74 (41)	89 (32)	158 (51)	44 (20)	30 (33)	
BCP-ALL Intermediate Risk (%)	827 (34)	489 (35)	66 (37)	82 (30)	87 (28)	89 (41)	14 (16)	
BCP-ALL High Risk (%)	818 (33)	478 (34)	39 (22)	104 (38)	67 (21)	84 (39)	46 (51)	
**EOI assessment**^a^	2540	1318	195	316	343	259	109	<0·0001
CR1 (%)	2458 (97)	1281 (97)	189 (97)	305 (99)	340 (99)	237 (92)	106 (97)	
**EOI MRD (BCP-ALL)**	2166	1281	166	237	255	143	84	0·0007
≥0·01%	497 (23)	318 (25)	19 (11)	50 (20)	50 (20)	43 (30)	17 (20)	
**EOI risk stratification**	2277	1286	168	250	293	193	87	<0·0001
BCP-ALL Standard Risk (%)	593 (26)	289 (22)	61 (36)	57 (23)	134 (46)	28 (15)	24 (28)	
BCP-ALL Intermediate Risk (%)	626 (28)	364 (28)	58 (35)	65 (26)	68 (23)	59 (31)	12 (14)	
BCP-ALL High Risk (%)	1058 (46)	633 (49)	49 (29)	128 (51)	91 (31)	106 (55)	51 (59)	
**Induction**								
Induction death (%)	127 (5)	64 (5)	7 (3)	24 (7)	19 (5)	10 (4)	3 (3)	0·2518
Induction failure (%)	82 (3)	37 (3)	6 (3)	11 (3)	3 (1)	22 (8)	3 (3)	0·0016
Treatment abandonment (%)^b^	7 (0·3)	4 (0)	1 (0)	0 (0)	1 (0)	1 (0)	0 (0)	0·8863
**Post induction**	2475	1286	193	307	341	241	107	
Deaths (%)	158 (6)	70 (5)	4 (2)	23 (8)	41 (12)	9 (4)	11 (10)	<0·0001
Relapse (%)	627 (25)	266 (21)	72 (37)	133 (43)	62 (18)	64 (27)	30 (28)	<0·0001
Treatment abandonment (%)	83 (3)	49 (4)	1 (1)	13 (4)	11 (3)	2 (1)	7 (7)	0·0026

BCP-ALL: B-precursor acute lymphoblastic leukaemia; CNS, central nervous system; CR1: first remission; EOI, end of induction.

IQR: interquartile range; MRD, minimal (measurable) residual disease.

PPR: poor prednisolone response; WBC, highest presentation white blood cell count.

p value for age and WBC analysed using Kruskall Wallis test; for the rest, chi-square test of independence.

a While 2557 patients reached EOI, CR1 information was not available for 17 patients.

b Four patients, lost to follow up were not included in the analysis.

At a median follow up of 61 (59–62) months, 1596 (59%)/2695 patients were in CR1 (Fig. 1). Of 285 (11%) treatment related deaths (TRD), 127 (45%) occurred during induction and 103 (36%) during the maintenance phase (Table 2). Induction TRD was lowest in provisional SR patients (cumulative incidence, 2%; 95% CI, 1·2–3·1%) compared to provisional IR (4%; 2·8–5·5%), HR (4·2%; 2·9–5·7%) and T-ALL (3·9%; 1·9–7%) patients (Gray test, p = 0·0023; Supplementary Figure S1). No significant differences in TRD were observed among risk-groups post-induction, including during maintenance (Table 2). At time of reporting, 627/2475 (25%) patients have relapsed post-remission (Table 3). The 4-year cumulative incidence of relapse for the entire cohort was 27% (26–29) and for SR, 21% (17–24); IR, 24% (21–28); HR, 33% (30–36) and T-ALL, 27% (21–34) (p < 0·0001) (Supplementary Figure S2) The majority (82%) of relapses were either very-early (243/627, 39%) or early (269/627, 43%) (Table 3). 461 (74%)/627, involved the bone marrow (isolated and combined) and isolated CNS relapses occurred in 133 (21%) (Table 3; Supplementary Tables S5, S6).

**Table 2:** Induction and post-induction events.

	Induction				Post-induction				
					Deaths (%)				
	**N**	Events	Deaths	Failure	**N**	Events	Intensive	Maintenance	Relapse
All patients	2695	209	127 (5)	82 (3)	2475	785	55 (2)	103 (4)	627 (25)
BCP-ALL SR	821	30	20 (2)	10 (1)	593	145	6 (1)	19 (3)	120 (20)
BCP-ALL IR	827	57	45 (5)	12 (1)	626	185	18 (3)	25 (4)	142 (23)
BCP-ALL HR	818	93	46 (6)	47 (6)	1058	395	27 (3)	54 (5)	314 (30)
T-ALL	229	29	16 (7)	13 (6)	198	60	4 (2)	5 (3)	51 (26)

BCP-ALL, B cell-precursor acute lymphoblastic leukaemia; SR, standard risk; IR, intermediate risk; HR, high risk.

B-precursor ALL risk groups. χ^2^ test (a) post-induction intensive phase deaths, p value: 0·0576, (b) maintenance phase deaths, p value 0·1552.

There were 11 patients excluded from the analysis who died post treatment for unknown cause.

**Table 3:** Distribution of relapses by timing, site and treatment centre (ICiCLe-ALL multicentre treatment protocol).

	All	Center 1	Center 2	Center 3	Center 4	Center 5	Center 6
	n (%)	n (%)	n (%)	n (%)	n (%)	n (%)	n (%)
**N**	2475	1286	193	307	341	241	107
**Time-point of relapse**							
Very early	243 (39)	88 (33)	16 (22)	64 (48)	30 (48)	28 (44)	17 (57)
Early	269 (43)	138 (52)	34 (47)	45 (34)	23 (37)	21 (33)	8 (27)
Late	115 (18)	40 (15)	22 (31)	24 (18)	9 (15)	15 (23)	5 (17)
**Type of relapse**							
**Isolated extramedullary**	166 (26)	54 (20)	30 (42)	43 (32)	16 (26)	15 (23)	8 (27)
CNS	133 (21)	46 (17)	16 (22)	39 (29)	13 (21)	14 (22)	5 (17)
Testis	29 (5)	8 (3)	13 (18)	4 (3)	3 (5)	0	1 (3)
CNS + Testes	1 (0·2)	0	1 (1)	0	0	0	0
Other	3 (0·5)	0	0	0	0	1 (2)	2 (7)
**Isolated medullary**	298 (48)	127 (48)	23 (32)	64 (48)	34 (55)	33 (52)	17 (57)
**Combined**	163 (26)	85 (32)	19 (26)	26 (20)	12 (19)	16 (25)	5 (17)
CNS	114 (18)	65 (24)	8 (11)	18 (14)	4 (6)	14 (22)	5 (17)
Testis	38 (6)	17 (6)	9 (13)	5 (4)	6 (10)	1 (2)	0
CNS + Testes	7 (1)	3 (1)	1 (1)	3 (2)	0	0	0
Other	4 (1)	0	1 (1)	0	2 (3)	1 (2)	0
**Total relapses**	**627 (25)**	**266 (21)**	**72 (37)**	**133 (43)**	**62 (18)**	**64 (27)**	**30 (28)**

CNS, central nervous system; very early relapse: relapse ≤18 months from diagnosis; early relapse: relapse >18 months from diagnosis but ≤6 months from end of treatment; late relapse: relapse >6 months from end of treatment. Combined: relapse involving bone marrow (medullary) and extramedullary sites.

**Fig. 2:**
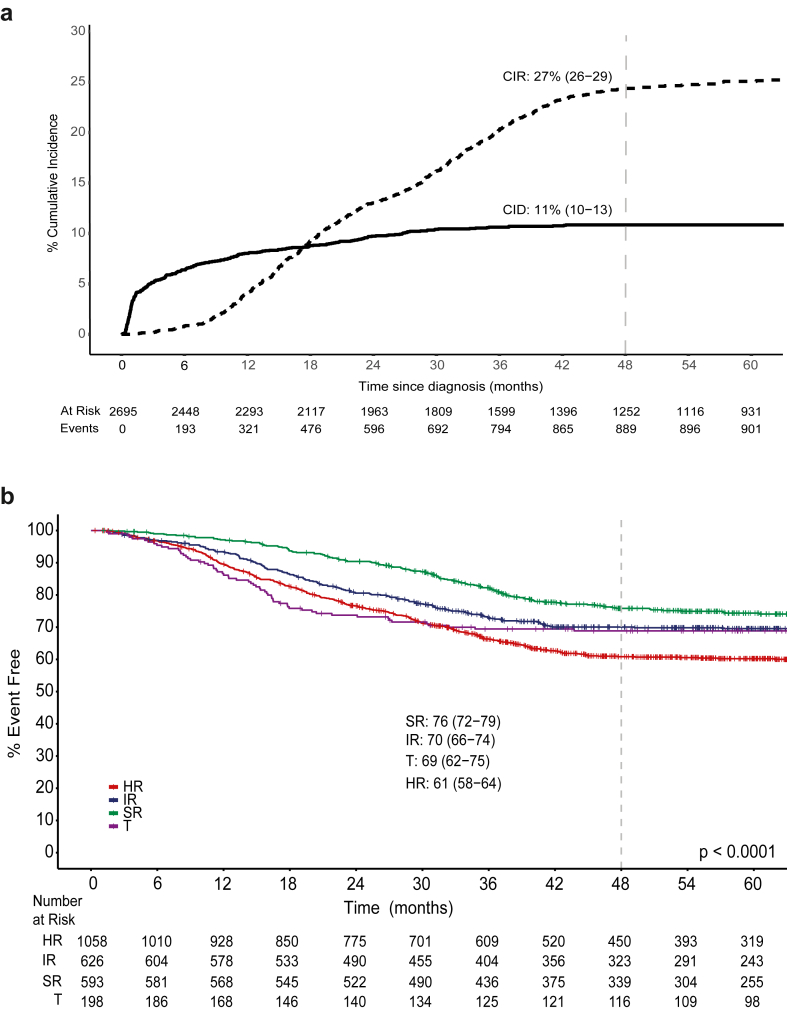
a: Cumulative incidences of relapse (CIR) and treatment related death (CID) in children with acute lymphoblastic leukaemia treated with the risk-stratified ICiCLe-2014 protocol. CID includes death in induction and death in CCR. Curves were constructed treating relapse and post-remission deaths as competing risks. Values indicate 48-month point estimates with 95% confidence intervals; p values were obtained using the Gray test for comparison of cumulative incidence estimates; b: Event free survival of patients risk stratified at day 35. SR, IR and HR, BCP-ALL standard, intermediate and high-risk respectively; T, T-ALL.

The cumulative incidences of death and relapse were 11% (10–13) and 27% (26–29) respectively (Fig. 2a). The 4-year EFS and OS for the entire cohort estimated from the time of diagnosis was 62% (95% CI, 60–64) and OS 74% (73–76) (Table 4). Outcomes in T- and BCP-ALL were respectively: 4Y-EFS, 60% (53–66) and 62% (60–64) (p = 0·1920); 4Y-OS, 69% (63–75) and 75% (73–77) (p = 0·453). Survival varied significantly by final risk group with 4-year EFS and OS estimated from end of induction as 76% (72–79%) and 88% (85–90%) in SR patients; 70% (66–74%) and 80% (77–83%) in IR patients; 61% (58–64%) and 73% (70–76%) in HR patients; 69% (62–75%) and 77% (70–83%) in T-ALL (p < 0·0001 for both EFS and OS) (Table 4; Fig. 2b and Supplementary Figure S3). Cox multivariable regression confirmed the independent prognostic influence of age, presentation WBC count, bulky disease, high-risk genetics and EoI-MRD levels, but not prednisolone response or CNS disease, on survival outcomes in BCP-ALL (Table 5).

**Table 4:** Univariate analysis (log-rank test) of risk factors influencing event-free (EFS) and overall survival (OS) of patients treated on the ICiCLe-ALL multicentre protocol.

	N	EFS (%)	95% CI	p value	OS (%)	95% CI	p value
**Overall**	2695	62%	60–64		74%	73–76	
**NCI risk**	2694						
Standard risk	1604	68%	66–70	<0·0001	80%	78–82	<0·0001
High risk	1090	54%	51–57		66%	63–69	
**Initial risk groups**	2695						
BCP-ALL standard risk	821	71%	68–74	<0·0001	84%	81–86	<0·0001
BCP-ALL intermediate risk	827	63%	59–66		73%	70–76	
BCP-ALL high risk	818	53%	50–57		67%	63–70	
T-ALL	229	60%	53–66		69%	63–75	
**Prednisolone response**	2575						
Good	2129	65%	63–67	0·0008	77%	75–79	<0·0001
Poor	446	53%	48–58		67%	62–72	
**Cytogenetics groups (BCP-ALL)**	2364						
High risk	271	45%	39–51	<0·0001	60%	53–66	<0·0001
High hyperdiploidy	606	73%	69–76		83%	80–86	
*ETV6::RUNX1*	239	67%	60–73		79%	73–84	
Others	1248	60%	58–63		73%	70–76	
**Post induction MRD (BCP-ALL)**	2166						
<0·01%	1669	70%	68–72	<0·0001	81%	79–83	0·0003
≥0·01%	497	51%	46–55		69%	65–73	
**Final risk groups**	2475						
BCP-ALL standard risk	593	76%	72–79	<0·0001	88%	85–90	<0·0001
BCP-ALL intermediate risk	626	70%	66–74		80%	77–83	
BCP-ALL high risk	1058	61%	58–64		73%	70–76	
T ALL	198	69%	62–75		77%	70–83	
**Lineage**	2695						
BCP-ALL	2466	62%	60–64	0·1920	75%	73–77	0·0453
T-ALL	229	60%	53–66		69%	63–75	
**Age**	2695						
<10 years	2051	64%	62–67	<0·0001	76%	75–79	<0·0001
≥10 years	644	55%	51–59		67%	63–70	
**Sex**	2695						
Male	1775	60%	57–62	0·0015	60%	57–62	0·215
Female	920	68%	64–70		67%	64–70	
**WC (x10**^9^**L)**	2692						
<50	2055	66%	64–68	<0·0001	78%	76–80	<0·001
≥50	637	49%	45–53		63%	59–67	
**Bulky disease**	2631						
Yes	808	58%	55–62	0·0062	71%	67–74	0·0074
No	1823	64%	61–66		76%	74–78	
**CNS disease**	2594						
Yes	87	52%	40–62	0·0009	64%	52–73	0·0008
No	2507	64%	62–66		76%	74–78	
**Centres**	2695						
Centre 1	1393	67%	65–70	<0·0001	77%	75–80	<0·0001
Centre 2	209	59%	52–65		78%	71–83	
Centre 3	342	44%	39–50		59%	53–64	
Centre 4	364	64%	59–69		78%	73–82	
Centre 5	274	63%	57–69		73%	67–78	
Centre 6	113	56%	46–65		72%	62–80	

BCP-ALL: B Cell Precursor Acute Lymphoblastic Leukemia; WC: Highest presenting white blood cell.

NCI: National Cancer Institute; Initial Risk: Risk stratification at day 8; CNS: Central Nervous System.

Final Risk: Risk stratification at end of induction; MRD: Minimal Residual Disease.

95% CI, 95% confidence interval.

**Table 5:** Multivariable regression analysis of factors influencing EFS, TRD and relapse in B cell-precursor ALL.

Covariates	Event-free Survival^a^			Overall Survival^a^			Treatment Related Death^b^			Relapse^c^		
	HR	95% CI	p value	HR	95% CI	p value	SHR	95% CI	p value	SHR	95% CI	p value
Male sex	1·30	1·10–1·54	**0·0017**	1·12	0·91–1·38	**0·2905**	1·08	0·72–1·61	0·724	1·33	1·10–1·62	**0·0041**
Age at diagnosis (years)	1·04	1·03–1·06	**<0·0001**	1·04	1·02–1·07	**0·0005**	0·96	0·90–1·02	0·159	1·05	1·03–1·07	**<0·0001**
WBC at presentation (×10^9^/L)	1·23	1·16–1·30	**<0·0001**	1·21	1·13–1·30	**<0·0001**	1·24	1·07–1·43	**0·0038**	1·19	1·12–1·27	**<0·0001**
Bulky disease	1·38	1·17–1·64	**0·0002**	1·4	1·13–1·74	**0·0025**	1·48	0·95–2·31	0·0807	1·38	1·13–1·68	**0·0014**
High risk cytogenetics (B-Cell)	1·55	1·25–1·91	**<0·0001**	1·56	1·19–2·04	**0·0012**	1·17	0·67–2·05	0·590	1·62	1·26–2·07	**0·0001**
Poor prednisolone response	1·13	0·92–1·34	0·242	1·03	0·80–1·34	0·8063	0·94	0·57–1·55	0·803	0·99	0·77–1·29	0·952
CNS disease	1·49	0·96–2·31	0·075	1·42	0·83–2·43	0·2022	1·74	0·64–4·72	0·274	0·97	0·52–1·82	0·921
EoI MRD ≥0·01%	1·90	1·62–2·23	**<0·0001**	1·55	1·26–1·91	**<0·0001**	1·26	0·83–1·92	0·287	1·47	1·22–1·79	**<0·0001**
**Treatment centre**												
Centre 2	1·91	1·47–2·48	**<0·0001**	1·55	1·09–2·19	**0·0149**	0·43	0·13–1·36	0·149	2·33	1·79–3·03	**<0·0001**
Centre 3	2·17	1·73–2·73	**<0·0001**	2·13	1·60–2·85	**<0·0001**	0·75	0·32–1·76	0·509	2·76	2·14–3·55	**<0·0001**
Centre 4	1·15	0·88–1·51	0·315	1·2	0·85–1·70	0·31	2·95	1·81–4·81	**<0·0001**	0·75	0·53–1·08	0·122
Centre 5	1·39	1·05–1·83	**0·020**	1·52	1·09–2·12	**0·0137**	0·83	0·33–2·10	0·697	1·34	0·96–1·88	0·086
Centre 6	1·87	1·31–2·68	**0·0006**	1·76	1·08–2·86	**0·0233**	2·91	1·48–5·74	**0·0021**	1·60	1·02–2·50	**0·039**

HR, hazard ratio; SHR, subdistribution hazard ratio; 95% CI, 95% confidence interval.

CNS, central nervous system; EoI MRD, end of induction minimal residual disease; WBC, white blood cell count.

Age and WBC count were continuous variables; Centre 1 served as reference centre in regression models.

Bold font in Table 5 denotes where covariate is significant in multivariable regression analysis.

a Cox multivariable regression modelling of risk factors influencing (4-year) event-free and overall survival in B-precursor ALL.

b,c Fine–Gray competing risk modelling of factors influencing cumulative incidence of treatment related death and relapse.

**Fig. 3:**
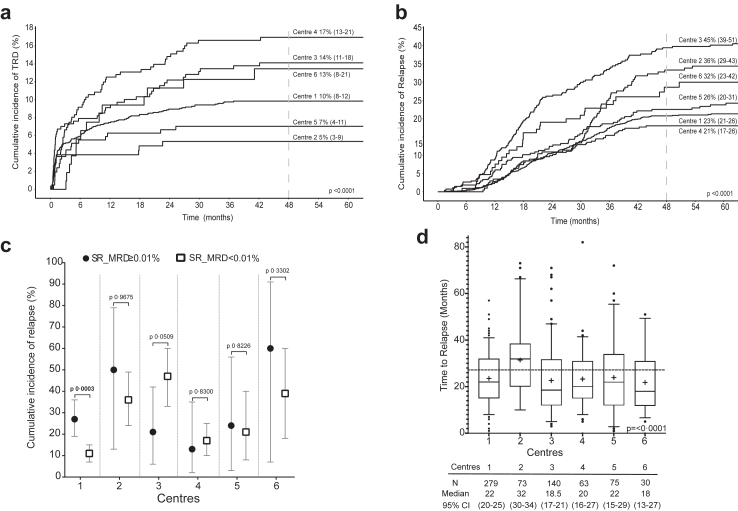
Cumulative incidence of treatment related deaths (TRD) (a) and relapse (b) at individual ICiCLe centres. Curves were constructed treating relapse and post-remission deaths as competing risks. Values indicate 48-month point estimates with 95% confidence intervals; p values were obtained using the Gray test for comparison of cumulative incidence estimates; c: Vertical forest plot representation of the cumulative incidence of relapse (estimated at 48 months, with 95% confidence interval, y-axis) in Standard Risk B cell-precursor ALL patients treated at individual centres. ∗∗ represents p value < 0·001 (Gray test for comparison of cumulative incidence estimates). d: Box and whisker plot showing median (horizontal line) and mean (+) time to relapse at the ICiCLe centres. Whiskers are at 95% confidence intervals and **·** are outliers. p value obtained using the Kruskal–Wallis test.

Multivariable Cox and competing-risk regression models showed treatment centre as an independent variable influencing survival, relapse and TRD (Table 5). Significant differences were observed among centres in rates of TRD, relapse and survival outcomes (Table 3 and Supplementary Tables S5, S7, S8). At 3 centres, the cumulative incidence of TRD approached or exceeded 10% (Fig. 3a, Supplementary Table S9). The 4-year cumulative incidence of relapse varied 2-fold among centres, from 21% to 45% (Fig. 3b, Supplementary Table S9). Consequently, a near 1·5-fold difference in 4-year EFS (44–67%) and OS (59%–78%) was observed among centres (p < 0·0001) (Table 4). Differences were observed among centres even after accounting for variations in patient demographics and disease characteristics. As example, Centres 3 and 5 with comparable patient profiles reported differences in treatment response (EoI CR, 99% versus 92%; EoI-MRD^hi^, 20% versus 30%), TRD (induction deaths 7% versus 4%; post-induction deaths, 8% versus 5%) and rates of relapse (43% versus 27%) (Table 1). Overall, in patients with non-high risk ALL, the influence of EoI-MRD on the cumulative incidence of relapse was restricted to patients with provisional intermediate risk ALL (Supplementary Table S10). Centre 1 alone demonstrated the prognostic influence of EoI-MRD in patients with provisional SR ALL (Fig. 3c). Centre 2 uniquely reported significantly late-onset relapse (p = 0·0001) (Fig. 3d) with significantly (p < 0·0001) higher proportion of patients with isolated extramedullary disease, particularly isolated testicular relapse (Table 3).

## Discussion

We report on a large cohort of children with ALL treated systematically across multiple centres in India (Supplementary Table S11). As a consortium,^9^ we first developed a consensus treatment protocol, introducing genetic and MRD stratification for children with ALL in India. With centres joining the consortium at different timepoints, a composite analysis of experience gathered over 6-years is presented here. Centres invested considerable efforts during this time to establish standards. With a requirement to complete genetic risk stratification by day 8, a triple probe FISH approach was introduced across laboratories, complemented with DNA ploidy assessment using flow cytometry. Centres improved MRD assessment over time.^10^^,^^11^ Workshops and training programs were held for all participating laboratories. For some centres, coming from BFM based protocols, there was an initial reluctance to treat BCP-ALL patients as SR as it was viewed to lack the required intensity. Centres lacking ability to measure serum methotrexate levels introduced a strategy of increased hydration and folinic acid rescue to avoid toxicity,^12^ but potentially with an increased risk of relapse. With patients often arriving unwell, the ICiCLe strategy was to start with a week of prednisolone, avoid the day 1 intrathecal therapy, improve the nutritional support and with the help of NGO's provide support for the families. This contributed to the low abandonment rate. With a common protocol, families were able to move back to centres closer to home and continue the same protocol. With time, centres were able to follow risk stratification on day 8 with EoI MRD assessed in 96% of eligible patients The improved survival rates in SR patients (EFS 76% and OS 88%) justified the very cost beneficial^13^ risk stratified approach to therapy for childhood ALL in low-middle-income countries (LMIC). This allowed us subsequently to launch a funded, randomized clinical trial ICiCLe ALL-14/InPOG-ALL-15 (CTRI/2015/12/006434).^7^

Though data was collected prospectively, due to initial lack of funding, we were unable to obtain data on toxicity other than deaths. While deaths in inductions decreased at all centres, overall TRD's ranged from 6 to 17%. Increased TRD's in children with cancer, particularly in childhood ALL, at centres in LMIC's have been reported,^14^ though reasons are unclear. In this study, induction TRD were significantly (p = 0·0023) lower in SR, who do not receive anthracyclines during this phase, compared to all other risk groups. SR patients additionally did not receive cyclophosphamide/cytarabine/6-mercaptopurine in consolidation. The differences in TRD between the centres was not investigated. All centres in this study are tertiary referral centres. Most families are poor and have visited at least one other hospital prior to obtaining definitive treatment. An increase in travel time to hospital is associated with more advanced disease at diagnosis^15^ and poorer outcomes,^16^ particularly in poorer families.^17^^,^^18^ Centres 1, 3, and 6 receive patients from all over the country. Centres 2 and 5 serve a wide rural catchment area. Centres 1, 2 and 5, with the lowest TRD's are situated within tertiary care cancer centres. Centres 3, 4, and 6 are within large tertiary general hospitals. Informal discussion with colleagues suggest that bed availability and rapid access to antibiotics within the “golden hour” for patients with febrile neutropenia^18^ are perhaps better at tertiary care cancer centres. Some patients live in remote areas where the nearest medical facility may involve an overnight journey. This is a problem during maintenance therapy where patients move back home. This was a major problem during the lockdown phase of the pandemic where deaths and relapses resulted from no access to drugs or medical facilities. Formal evaluation of how factors that influenced treatment-related toxicities are beyond the scope of this paper and a new ICiCLe Implementation Study (CTRI/2023/12/060828) is in process to identify potential causative factors and solutions. An additional limitation is the study's focus on patients receiving treatment at select centres. Thus the observations may not be an accurate reflection of real-world data.

Relapse rates of 21–43% across centres were disappointing. EFS for BCP-ALL SR and IR patients in our study were 76% and 70% respectively and lower than expected. The treatment protocol for SR patients is similar to that used in UKALL 2003 with a reported 5-year EFS of 95%^19^ and the Children's Oncology Group (COG) AALL0331 trial with a reported 6-year EFS of 91%.^20^ One possible explanation is that the genetic risk stratification does not include the more recently reported Ph-like or the *IKZF1* deleted subtypes. These are diagnosed by RNA sequencing and/or copy number evaluation, require intensive and/or targeted therapy and were possibly classified as SR.^21^ However, this was true of the COG and UKALL studies as well.

There was considerable variation in MRD positivity at the centres, which improved with time, Centre 2, with a MRD positive rate of 11% had relapse rates were 37%, but centres 1 and 5 with MRD positive rates of 25% and 30%, reported relapse rates of 25% and 27% respectively. For SR patients treated with a 3-drug regimen, the COG using FCM-MRD reported 80% with MRD <0·01%,^20^ comparable to the 75% reported in these analyses. However, in the COG cohort the incidence of isolated extramedullary relapse and marrow involvement were 2% and 5·3%, compared to 5% and 16% in the ICiCLe SR cohort respectively. Furthermore, in the ICiCLe cohort, relapses in SR/IR patients irrespective of MRD levels primarily occurred while patients were still on treatment, mostly involving the marrow suggesting insufficient therapy for all patients. For SR patients, COG and UKALL protocols used dexamethasone instead of prednisolone and polyethylene glycol (PEG) conjugated ASNase while ICiCLe centres used biogeneric native ASNase. Inferior ASNase products (widely available in India)^22^ and inadequate ASNase therapy^23^ significantly contribute to increased relapse, particularly at extramedullary sites.^24^ Centre 2 used a single ASNase biosimilar shown to be ineffective for most of this study period,^25^ while other centres used a mixture of brands. Possibly this contributed to the increase in extramedullary relapses observed at Centre 2. Therapeutic drug monitoring and identification of more suitable ASNase have been introduced and drug companies persuaded to remove unsuitable products from the market. Intensification of 6-Mercaptopurine (6-MP) dose is another issue as children in India have a higher prevalence of *NUDT15* polymorphisms^26^ with lower tolerance and 36% of TRD's occurred in maintenance. Until recently only 50 mg scored tablets were available. Working with pharmaceutical companies, 10 mg tablets and a liquid formulation (10 mg/ml) have been introduced allowing therapeutic optimisation for younger children and for slow metabolisers.^27^ With systematic close monitoring the weighted mean dose of 6-MP during maintenance can be successfully increased without increasing toxicity.^28^^,^^29^

Disease-specific guidelines as a starting point for developing national strategies with collaborative research initiatives are the first step to reduce TRD.^9^ Enrolling children into multicentre clinical trials potentially decreases relapses and improves outcomes across centres. The experience of this consortium shows that adoption of a successful treatment strategy developed in high income countries does not readily translate into comparable results. It takes time and repeated training to achieve uniformity. With the ICiCLe network poised to increase to ∼40 centres covering most of the country in a hub-and-spoke model over the next 2-years, we have begun a series of workshops to ensure uniformity in diagnosis, monitoring (MRD) and data reporting. Newer challenges are emerging. RNA sequencing and analyses of copy number changes for genetic risk stratification are being introduced. Next generation sequencing for MRD allows standardisation across all centres and better discrimination of SR patients with a very low probability of relapse.^30^ Potentially these tests increase costs and benefit from centralisation of resources so that they are available to all patients and not just those at a few elite centres. With increasing expertise at secondary centres, TRD in the maintenance phase can be reduced through better access and development of shared care with regional hospitals. Automated software solutions to provide decision support during maintenance therapy are being developed. Our experience shows that along with the common challenges to improving outcomes of patients with cancer LMIC's, there are likely to be those peculiar to geographical health regions. Identification of these issues require multicentre collaboration, systematic treatment and data collection and a continuing dialogue with health care providers, non-government organizations and industry.

## Contributors

AT, GN, RS, SaB, SB, SK, VR and VS conceptualised the study, developed methodology and enrolled patients; PT, MS, AC and MP performed laboratory tests; MG, PD, ND, SD, VS and SK curated and analysed data. MG, PD, VS and SK wrote the paper. SK provided supervision for the study and VS was responsible for funding. All authors reviewed the final draft and took the decision to submit the manuscript.

## Data sharing statement

Beginning 3 months after and ending 36 months after publication, individual participant data that underlie the results reported in this article after deidentification will be available from the corresponding authors. Study protocol and statistical plan are already available. Post 36 months, data will be available on the institution server and accessed via ttcrc.org but without investigator support other than deposited metadata. Data will be provided to researchers who provide a methodologically sound proposal to enhance the aims of the study which has been approved by an independent review board.

## Declaration of interests

Authors do not have any conflict of interest to declare.
