# Supplementary material for: Risk stratified treatment for childhood acute lymphoblastic leukaemia: a multicentre observational study from India
Source: Lancet Reg Health Southeast Asia. 2025 May 13;37:100593. doi: 10.1016/j.lansea.2025.100593 (PMC12142348; doi:10.1016/j.lansea.2025.100593)
Supplement: Supplementary Figures and Tables [file mmc1.docx]

**Supplementary Material**

Uncovering key barriers to improving acute lymphoblastic leukaemia outcomes: the Indian Collaborative Childhood Leukaemia Study Group (ICiCLe) experience

MP Gogoi, P Das, N Das *et al*

Contents

[Tables S1A-C: ALL Risk stratification, ALL Risk Groups, ICiCLe-ALL Risk-stratified treatment 4](#_Toc194417319)

[Table S1A 4](#_Toc194417320)

[Table S1B 5](#_Toc194417321)

[Table S1C: 6](#_Toc194417322)

[Table S2: Discretionary provisional risk stratification in B cell-precursor ALL patients with missing information at day 8 (296/2466 patients) 8](#_Toc194417323)

[Table S3: B cell-precursor ALL, Final risk stratification 9](#_Toc194417324)

[Table S4: Missing data for key variables in the ICiCLe-ALL multicentre study 9](#_Toc194417325)

[Table S5: Timing of relapses by ICiCLe-ALL protocol treatment phase 10](#_Toc194417326)

[Table S6: Relapses within risk groups, time and site of relapse 10](#_Toc194417327)

[Table S7: Treatment-related deaths reported by centres at different treatment phases 11](#_Toc194417328)

[Table S8A-B 11](#_Toc194417329)

[Table S8A: EFS of patients, by treatment centre 11](#_Toc194417330)

[Table S8B: Pairwise EFS comparisons between centres (log-rank test) 11](#_Toc194417331)

[Table S9: Data for Figures 12](#_Toc194417332)

[Table S10: Cumulative incidence of relapse in provisional non-high risk B cell-precursor acute lymphoblastic leukaemia patients (BCP-ALL), stratified by levels of bone marrow minimal residual disease at end of induction (EoI-MRD). 13](#_Toc194417333)

[Table S11: Outcomes of previous treatment protocols for newly diagnosed paediatric acute lymphoblastic leukaemia in India 14](#_Toc194417334)

[Supplementary Figure Legends 15](#_Toc194417335)

[Figure S1 16](#_Toc194417336)

[Figure S2 17](#_Toc194417337)

[Figure S3 18](#_Toc194417338)

**Data analysis using R**

Analysis was carried out in the R statistical environment using R software version 4.4.1. R packages were used to read Microsoft Excel files (‘readxl’^1^), perform descriptive statistical analyses and hypothesis-testing (‘EpiR’^2^), analyse survival outcomes (‘survival’^3^) and evaluate factors influencing the same (‘coxphf’^4^ for Cox multivariable regression modelling), perform competing risk modelling of relapse and treatment-related death (‘cmprsk’^5^) and visualise findings using ‘ggplot2’^6^ and related data visualisation packages (‘survminer’^7^ and ‘ggsurvfit’^8^ for Kaplan-Meier curves; ‘tidycmprsk’^9^ for competing risk data; ‘ggpubr’^10^, ‘patchwork’^11^, ‘cowplot’^12^ and ‘gridExtra’^13^ for preparation of publication-suitable images)

1. https://cran.r-project.org/web/packages/readxl/index.html
2. https://cran.r-project.org/web/packages/survival/index.html
3. https://cran.r-project.org/web/packages/survminer/index.html
4. https://cran.r-project.org/web/packages/coxphf/index.html
5. https://cran.r-project.org/web/packages/cmprsk/index.html
6. https://cran.r-project.org/web/packages/epiR/index.html
7. https://cran.r-project.org/web/packages/tidycmprsk/index.html
8. https://cran.r-project.org/web/packages/ggsurvfit/index.html
9. https://cran.r-project.org/web/packages/ggplot2/index.html
10. https://cran.r-project.org/web/packages/ggpubr/index.html
11. https://cran.r-project.org/web/packages/patchwork/index.html
12. https://cran.r-project.org/web/packages/cowplot/index.html
13. https://cran.r-project.org/web/packages/gridExtra/index.html

**Cytogenetic characterisation of ALL samples in the ICiCLe ALL study**

Diagnostic bone marrow aspirate samples were subjected to both fluorescence in-situ hybridisation (FISH) analysis (using a 4-probe panel) and flow-based DNA ploidy analysis, with or without karyotyping^1^. The FISH panel included fusion probes to identify *ETV6*::*RUNX1* and *BCR*::*ABL1* fusions and break-apart probes to identify *KMT2A* and *TCF3* rearrangements. The gene partner in *TCF3* rearrangements was confirmed using results from karyotyping or by using a *TCF3*/*PBX1*/*HLF* tricolour fusion probe.

Aneuploidies were characterised using flow cytometry-based DNA ploidy analysis, corroborated where available by karyotyping and FISH studies. DNA ploidy analysis was performed using FxCycle™ Violet DNA staining as described previously^2^. DNA index was calculated as a ratio of the geometric means of FxCycle^TM^ Violet dye staining intensities in G0/G1 peaks of precursor B-lymphoblasts (identified using scatter properties and expression patterns of CD10, CD19 and CD45 antigens) and normal lymphocytes. A DNA index ≥ 1·16 indicated high hyperdiploidy. In these patients, FISH analysis with the *ETV6*::*RUNX1* fusion probe often revealed extra discrete *RUNX1* signals, and additional FISH analysis using centromeric probes typically revealed trisomies of chromosomes 4, 10, and 17. A DNA index ≤ 0·70 indicated hypodiploidy (including the low hypodiploid and near-haploid subtypes), corroborated by additional FISH analyses using probes targeting chromosomes 3, 7, 15 and 17 to identify the respective monosomies^3^. A DNA index ≥ 1·40 indicated near-triploidy.

Distribution of high risk BCP-ALL cytogenetic subtypes [271 (11·4%)/2364] in the ICiCLe-ALL observational study were as below-

- *BCR::ABL1*: 106 (4%)
- Hypodiploidy: 133 (6%)
- iAMP21: 3 (0·1%)
- *KMT2A* rearranged: 27 (1%)
- *TCF3::HLF*: 1 (0·04%)
- Not available: 1 (0·04%)

**References**

1. Parihar M, Singh MK, Islam R, Saha D, Mishra DK, Saha V, Krishnan S. A triple-probe FISH screening strategy for risk-stratified therapy of acute lymphoblastic leukaemia in low-resource settings. Pediatr Blood Cancer. 2018;65:e27366.
2. Gupta N, Parihar M, Banerjee S, Brahma S, Pawar R, Rath A, et al. FxCycle™ based ploidy correlates with cytogenetic ploidy in B-cell acute lymphoblastic leukemia and is able to detect the aneuploid minimal residual disease clone. Cytometry B Clin Cytom. 2019;96(3):239-248. doi: 10.1002/cyto.b.21765.
3. Gupta T, Arun SR, Babu GA, Chakrabarty BK, Bhave SJ, Kumar J, et al. A systematic cytogenetic strategy to identify masked hypodiploidy in precursor B acute lymphoblastic leukemia in low-resource settings. Indian J Hematol Blood Transfus. 2021;37(4):640-648. doi: 10.1007/s12288-021-01409-w.

# Tables S1A-C: ALL Risk stratification, ALL Risk Groups, ICiCLe-ALL Risk-stratified treatment

## Table S1A

| **Risk stratification criteria in the ICiCLe-ALL protocol** | |
| --- | --- |
| **At presentation** | |
| ALL lineage | B cell-precursor ALL or T-ALL |
| NCI Risk | *NCI Standard Risk* |
|  | Age < 10 years; presentation white cell count < 50 × 10^9^/L |
|  | *NCI High Risk* |
|  | Age ≥ 10 years and/or presentation white cell count ≥ 50 × 10^9^/L |
| Bulky disease | Enlarged peripheral lymph node(s), largest diameter ≥ 5 cm |
|  | Liver and/or spleen enlarged to umbilicus or beyond |
|  | Mediastinal mass ≥ 1/3 of intrathoracic diameter on chest x-ray (PA) |
|  | Testicular disease (clinical ± ultrasound diagnosis) |
| Cytogenetics | High-risk cytogenetics if any of the following: |
|  | *BCR*::*ABL1* (or other ABL-class) ALL |
|  | *KMT2A* rearranged ALL |
|  | Intrachromosomal amplification of chromosome 21 (iAMP21) |
|  | Hypodiploidy (modal chromosome number < 45 chromosomes) |
|  | *TCF3*::*HLF* ALL |
| CNS disease | CNS3 disease (CSF assessed on day 8) |
|  | CSF pleocytosis (≥ 5 leucocytes / µL) with unequivocal blasts |
|  | ± Clinical features indicating CNS disease (e.g., cranial nerve palsy) |
| Prior treatment | Any ALL-directed treatment prior to presentation at study centre |
|  |  |
| **At Day 8 (following 7 days of prednisolone monotherapy)** | |
| Prednisolone | • Good prednisolone response (GPR) |
| response | Peripheral blast count < 1000 / µL (< 1 × 10^9^/L) |
|  | • Poor prednisolone response (PPR) |
|  | Peripheral blast count ≥ 1000 / µL (≥ 1 × 10^9^/L) |
|  |  |
| **At Day 35 (end of the induction treatment phase)** | |
| Complete | Bone marrow blasts < 5% (in a regenerating marrow) |
| Remission (CR) | Satisfactory blood counts, no circulating blasts, no clinical disease |
|  | If CNS disease, clearance of CSF blasts |
|  | If mediastinal mass, mass regression by ≥ 35% on imaging |
| MRD | Bone marrow MRD, multiparameter flow cytometry, 8-10 antigens |
|  | Low (or Negative): MRD < 0·01% |
|  | High (or Positive): MRD ≥ 0·01% (or indeterminate / non-evaluable) |
| ALL, acute lymphoblastic leukaemia; CNS, central nervous system; CSF, cerebrospinal fluid; MRD, minimal residual disease; NCI, National Cancer Institute; PA, postero-anterior chest radiograph | |
| Prednisolone monotherapy, oral prednisolone 60 mg/m^2^/day, for 7 days | |
| ICiCLe, Indian Collaborative Childhood Leukaemia Study Group | |
| For FISH, initially 4 probes were used, *ETV6::RUNX1* fusion, *BCR::ABL1* fusion, *KMT2A* rearrangement and *TCF3::PBX1 fusion*. Subsequently *BCR::ABL1* break apart and ABL2 fusion probes were added | |

## Table S1B

| **T-ALL (including T-lymphoblastic lymphoma)** | | |
| --- | --- | --- |
| No risk stratification | | |
|  | | |
| **B cell-precursor ALL (BCP-ALL)** | | |
| **Initial Risk Groups (risk stratification at Day 8, following the prednisolone prephase)** | | |
| Standard Risk (SR) | NCI Standard Risk | |
|  | No bulky disease, no CNS disease, no prior treatment | |
|  | No high-risk cytogenetics | |
|  | Good prednisolone response | |
| Intermediate Risk (IR) | NCI High Risk and/or bulky disease | |
|  | No CNS disease, no prior treatment | |
|  | No high-risk cytogenetics | |
|  | Good prednisolone response | |
| High Risk (HR) | Any of the following | |
|  | - High risk cytogenetics |  |
|  | - CNS disease | |
|  | - Poor prednisolone response | |
|  | - Prior treatment | |
| **Final Risk Groups (risk stratification at Day 35, i.e. end of induction)** | | |
| Final SR | Initial standard risk features, accompanied by | |
|  | Complete remission at end of induction; and | |
|  | Low MRD | |
| Final IR | Initial intermediate risk features, accompanied by | |
|  | Complete remission at end of induction; and | |
|  | Low MRD | |
| Final HR | Initial SR and IR patients with | |
|  | Non-remission at end of induction; and/or | |
|  | High MRD | |
|  | All other patients with initial HR features | |
| CNS, central nervous system; NCI, National Cancer Institute; MRD, minimal residual disease | | |

Table S1C:

**ICiCLe-ALL treatment protocol, first-presentation acute lymphoblastic leukaemia, 1-18 years old**

| **Risk Group** | **Steroid Prophase** | | **Induction** | **Consolidation** | **Interim Maintenance** | | **Delayed Intensification** | **Maintenance** |  |  |
| --- | --- | --- | --- | --- | --- | --- | --- | --- | --- | --- |
| **Standard** | Days 1-7 | | Week 2 - 5 | Week 6 - 8 | Week 9 - 17 | | Week 18 - 24 | Week 25 - 120 |  |  |
| B-cell precursor ALL | Prednisolone 60 mg/m^2^ | | Prednisolone 60 mg/m^2^ | 6-mercaptopurine oral | 6-mercaptopurine oral | | Dexamethasone oral | In each 12 week cycle |  |  |
| And all of the following | oral | | oral, Day 8-28, & taper | 60 mg/m^2^, Days 1-21 | 60 mg/m^2^, Days 1-49 | | 10 mg/m^2^, Days 1-5 & | 6-mercaptopurine oral |  |  |
| NCI Standard Risk |  | | Vincristine 1·5 mg/m^2^ | IT Methotrexate | IT Methotrexate | | Days 15-19 | 60 mg/m^2^, Days 1-84 |  |  |
| Non-bulky disease |  | | IV, Day 8, 15, 22, 29 | Day 8, 15 | Days 15, 43 | | Vincristine 1·5 mg/m^2^ | Methotrexate 20 mg/m^2^ |  |  |
| No high risk genetics |  | | *E. coli* L-asparaginase |  | Methotrexate 20 mg/m^2^ | | IV, Day 1, 8, 15 | oral, once a week |  |  |
| No extramedullary sites |  | | IM, 10,000 IU/m^2^ |  | oral, Day 1, 8, 22, 29 | | Doxorubicin 25 mg/m^2^ | IT Methotrexate |  |  |
| of disease |  | | Day 18,21,24, 27 |  | Day 36, 50, 57 | | IV, Day 1, 8, 15 | once in 12 weeks (oral |  |  |
| Good prednisolone |  | | IT Methotrexate |  | Vincristine 1·5 mg/m^2^ | | *E. coli* L-asparaginase | methotrexate omitted |  |  |
| response |  | | Day 8, 15, 25 |  | IV, Day 1, 29 | | IM, 10,000 IU/m^2^ | on week of IT) |  |  |
| MRD <0·01% at end of |  | |  |  | Dexamethasone oral | | Day 4, 7, 10, 13 |  |  |  |
| induction |  | |  |  | 6 mg/m^2^, Days 1-5 & | | IT Methotrexate |  |  |  |
|  |  | |  |  | Days 29-33 | | Days 1, 15 |  |  |  |
|  |  | |  |  |  | | Cyclophosphamide IV |  |  |  |
|  |  | |  |  |  | | 1 g/m^2^, Day 29 |  |  |  |
|  |  | |  |  |  | | Cytarabine 75mg/m^2^ IV |  |  |  |
|  |  | |  |  |  | | Days 30-33; 37-40 |  |  |  |
|  |  | |  |  |  | | 6-mercaptopurine oral |  |  |  |
|  |  | |  |  |  | | 60mg/m^2^, Days 29-42 |  |  |  |
|  |  | |  |  |  | |  |  |  |  |
| **Intermediate** | Days 1-7 | | Week 2 - 5 | Week 6 - 10 | Week 11 - 18 | | Week 19 - 25 | Week 26 - 121 |  |  |
| B-cell precursor ALL | Prednisolone 60mg/m^2^ | | Prednisolone 60 mg/m^2^ | 6-mercaptopurine oral | Methotrexate IV | | As in Standard Risk | As in Standard Risk |  |  |
| And any of the following | oral | | oral, Day 8-28, & taper | 60 mg/m^2^, Days 1-28 | 100 mg/m^2^ Day 2 | |  |  |  |  |
| NCI High Risk |  | | (Age ≥ 10 years, pulse | Cyclophosphamide IV | Dose increased by | |  |  |  |  |
| Bulky disease |  | | Prednisolone Days 8-14 | 1 g/m^2^, Day 1, 15 | 50 mg/m^2^ in each | |  |  |  |  |
| Testicular disease |  | | & Days 22-28) | Cytarabine 75mg/m^2^ IV | of the next treatments | |  |  |  |  |
| And |  | | Vincristine 1·5 mg/m^2^ | Days 2-5; 9-12; 16-19 | Day 12, 22, 32, 42 | |  |  |  |  |
| No high risk genetics |  | | IV, Day 8, 15, 22, 29 | Days 23-26 | Vincristine 1·5 mg/m^2^ | |  |  |  |  |
| No CNS disease |  | | Daunorubicin 25 mg/m^2^ | IT Methotrexate | IV, Day 2, 12, 22, 32 | |  |  |  |  |
| Good prednisolone |  | | IV, Day 8, 15 | Day 8, 15 | Day 42 | |  |  |  |  |
| response |  | | *E. coli* L-asparaginase |  | IT Methotrexate | |  |  |  |  |
| MRD <0·01% at end |  | | IM, 10,000 IU/m^2^ |  | Day 1, 31 | |  |  |  |  |
| of induction |  | | Day 9, 12, 15, 18, 21 |  |  | |  |  |  |  |
|  |  | | Day 24, 27, 30 |  |  | |  |  |  |  |
|  |  | | IT Methotrexate |  |  | |  |  |  |  |
|  |  | | Day 8, 15, 25 |  |  | |  |  |  |  |
|  |  | |  |  |  | |  |  |  |  |
| **High** | | Days 1-7 | Week 2 - 5 | Week 6 - 14 | | Week 15 - 22 | Week 23 - 29 | Week 30 - 125 | |  |
| B-cell precursor ALL | | Prednisolone 60mg/m^2^ | Prednisolone 60 mg/m^2^ | 6-mercaptopurine oral | | Methotrexate IV 3 g/m^2^ | As in Standard Risk | As in Standard Risk | |  |
| And any of the following | | oral | oral, Day 8-28, & taper | 60 mg/m^2^, Days 1-14 | | Day 1, 15, 29, 43 |  |  | |  |
| High risk genetics | |  | Vincristine 1·5 mg/m^2^ | & Days 29-42 | | IT Methotrexate |  |  | |  |
| CNS disease | |  | IV, Day 8, 15, 22, 29 | Cyclophosphamide IV | | Day 1, 15, 29, 43 |  |  | |  |
| Poor prednsolone | |  | Daunorubicin 25 mg/m^2^ | 1 g/m^2^, Day 1, 29 | | 6-mercaptopurine oral |  |  | |  |
| response | |  | IV, Day 8, 15, 22, 29 | Cytarabine 75mg/m^2^ IV | | 25 mg/m^2^, Days 1-49 |  |  | |  |
| MRD ≥0·01% at end | |  | *E. coli* L-asparaginase | Days 2-5; 9-12; 30-33 | |  |  |  | |  |
| of induction | |  | IM, 10,000 IU/m^2^ | Days 37-40 | |  |  |  | |  |
|  | |  | Day 9, 12, 15, 18, 21 | Vincristine 1·5 mg/m^2^ | |  |  |  | |  |
|  | |  | Day 24, 27, 30 | IV, Day 16, 23, 44, 51 | |  |  |  | |  |
|  | |  | IT Methotrexate | *E. coli* L-asparaginase | |  |  |  | |  |
|  | |  | Day 8, 15, 25 (CNS | IM, 10,000 IU/m^2^ | |  |  |  | |  |
|  | |  | disease, extra doses) | Day 15, 18, 21, 24 | |  |  |  | |  |
|  | |  |  | Day 43, 46, 49, 52 | |  |  |  | |  |
|  | |  |  | IT Methotrexate | |  |  |  | |  |
|  | |  |  | Day 1, 8, 29 | |  |  |  | |  |
|  | |  |  |  | |  |  |  | |  |
| **T-lymphoblastic** | | Days 1-7 | Week 2 -5 | Week 6 - 14 | | Week 15 - 22 | Week 23 - 29 | Week 30 - 125 | |  |
| **leukaemia / lymphoma** | | Prednisolone 60mg/m^2^ | Dexamethasone oral | As in High Risk | | Methotrexate IV 5 g/m^2^ | As in Standard Risk | As in Standard Risk | |  |
|  | | Oral | 10mg/m^2^ Days 8-14 |  | | Day 1, 15, 29, 43 |  |  | |  |
|  | |  | & Days 22-28 |  | | Rest as in High Risk |  |  | |  |
|  | |  | (replaces Prednisolone) |  | |  |  |  | |  |
|  | |  | Rest as in High Risk |  | |  |  |  | |  |
|  | |  |  |  | |  |  |  | |  |
| ICiCLe, Indian Collaborative Childhood Leukaemia Study Group; NCI, National Cancer Institute, High Risk if presentation white blood cell count ≥ 50×10^9^/L and/or age ≥ 10 years at diagnosis | | | | | | | | | | |
| High risk cytogenetics, Hypodiploidy (<45 chromosomes); *BCR::ABL1* fusion, *KMT2A* rearrangement, intrachromosomal amplifcation of chromosome 21, *TCF3::HLF* fusion | | | | | | | | | | |
| CNS disease, cerebrospinal fluid pleocytosis (≥ 5 cells/microlitre) with unequivocal blasts on CSF cytospin; clinical features of CNS disease (e.g. cranial nerve palsies) | | | | | | | | | | |
| Bulky disease, liver and/or spleen enlarged to umbilicus or beyond; any single lymph node ≥ 5 cm in maximum diameter; mediastinal mass ≥ 1/3rd of intrathoracic diameter on chest radiograph | | | | | | | | | |  |
| Good prednisolone response, absolute circulating blast count < 1000 / microlitre (< 1 × 10^9^/L) after 7 days of prednisolone monotherapy | | | | | | | | | |  |
| MRD, minimal residual disease in bone marrow estimated by 8 or 10-colour flowcytometry cytometry | | | | | | | | | |  |
| IT, intrathecal (intrathecal methotrexate dose based on age; 1-2 years 8 mg; 2-3 years 10 mg; older than 3 years, 12 mg); IV, intravenous; IM, intramuscular | | | | | | | | | |  |
| Asparaginase, where feasible, 1 dose of intramuscular PEG-asparaginase 1000 IU/m^2^ replaces 4 doses of native *E. coli* L-asparaginase | | | | | | | |  | |  |
| In patients with CNS disease, 18 Gy cranial irradiation before start of maintenance treatment, no intrathecal treatments subsequently | | | | | | | |  | |  |
| Infection prophylaxis, Trimethoprim-Sulphamethoxazole (twice daily, 2 consecutive days in a week) through all treatment phases (omitted with high-dose methotrexate blocks) | | | | | | | | | | |

# Table S2: Discretionary provisional risk stratification in B cell-precursor ALL patients with missing information at day 8 (296/2466 patients)

|  |  |  |  |  |  |  |  |  |  |  |  |  |  |  |  |
| --- | --- | --- | --- | --- | --- | --- | --- | --- | --- | --- | --- | --- | --- | --- | --- |
|  | **All centres** | | Centre 1 | | Centre 2 | | Centre 3 | | Centre 4 | | Centre 5 | | Centre 6 | | *p* |
|  | N | % | N | % | N | % | N | % | N | % | N | % | N | % |  |
| **Treatment response** |  |  |  |  |  |  |  |  |  |  |  |  |  |  | <0·0001 |
| **Day 8 risk stratification** | 2466 |  | 1393 |  | 179 |  | 275 |  | 312 |  | 217 |  | 90 |  |  |
| Missing information | 296 | 12 | 146 | 10 | 18 | 10 | 67 | 24 | 39 | 13 | 15 | 7 | 11 | 12 |  |
| No missing variables | 2170 | 88 | 1247 | 90 | 161 | 90 | 208 | 76 | 273 | 88 | 202 | 93 | 79 | 88 |  |
| **Day 8 risk stratification** |  |  |  |  |  |  |  |  |  |  |  |  |  |  |  |
| Standard Risk | 735 |  | 387 |  | 72 |  | 68 |  | 139 |  | 41 |  | 28 |  |  |
| Allocation off protocol | 3 | 0.4 | 0 | 0 | 1 | 1 | 2 | 3 | 0 | 0 | 0 | 0 | 0 | 0 |  |
| Allocation per protocol | 732 | 99.6 | 387 | 100 | 71 | 99 | 66 | 97 | 139 | 100 | 41 | 100 | 28 | 100 |  |
| Intermediate Risk | 735 |  | 449 |  | 57 |  | 59 |  | 74 |  | 84 |  | 12 |  |  |
| Allocation off protocol | 8 | 1 | 3 | 1 | 1 | 2 | 3 | 5 |  | 0 |  | 0 | 1 | 8 |  |
| Allocation per protocol | 727 | 99 | 446 | 99 | 56 | 98 | 56 | 95 | 74 | 100 | 84 | 100 | 11 | 92 |  |
| High Risk | 700 |  | 411 |  | 32 |  | 81 |  | 60 |  | 77 |  | 39 |  |  |
| Allocation off protocol | 24 | 3 | 0 | 0 | 4 | 13 | 4 | 5 | 0 | 0 | 11 | 14 | 5 | 13 |  |
| Allocation per protocol | 676 | 97 | 411 | 100 | 28 | 88 | 77 | 95 | 60 | 100 | 66 | 86 | 34 | 87 |  |
| Overall | 2170 |  | 1247 |  | 161 |  | 208 |  | 273 |  | 202 |  | 79 |  |  |
| Allocation off protocol | 35 | 2 | 3 | 0 | 6 | 4 | 9 | 4 |  | 0 | 11 | 5 | 6 | 8 |  |
| Allocation per protocol | 2135 | 98 | 1244 | 100 | 155 | 96 | 199 | 96 | 273 | 100 | 191 | 95 | 73 | 92 |  |
| Risk stratification variables include highest presentation white cell count, bulky disease status, prednisolone response, central nervous system disease, and cytogenetic subtype | | | | | | | | | | | | | | | |
| (p value, chi-square test of independence) | | |  |  |  |  |  |  |  |  |  |  |  |  |  |

| Table S3: B cell-precursor ALL, Final risk stratification | | | | | | | | | |
| --- | --- | --- | --- | --- | --- | --- | --- | --- | --- |
|  | | | | | | |  |  |  |
| **Day 8 Risk** | **Provisional SR** | | | **Provisional IR** | | | **High Risk** | | |
| Patients |  | 791 |  |  | 766 |  |  | 720 |  |
| EoI-MRD | Low | High | NA | Low | High | NA | Low | High | NA |
|  | 575 | 189 | 27 | 590 | 139 | 37 | 504 | 169 | 47 |
| **Final Risk** |  |  |  |  |  |  |  |  |  |
| SR | 572 | · | 21 | · | · | · | · | · | · |
| IR | 1 | · | 3 | 589 | · | 33 | · | · | · |
| HR | 2 | 189 | 3 | 1 | 139 | 4 | 504 | 169 | 47 |
| SR, Standard Risk; IR, Intermediate Risk; HR, High Risk B cell-precursor ALL | | | | | | | | |  |
| EoI, end of induction; MRD Low, <0·01%, High ≥ 0·01%; NA, not available | | | | | | | | | |

| Table S4: Missing data for key variables in the ICiCLe-ALL multicentre study | | | | | | | | | |
| --- | --- | --- | --- | --- | --- | --- | --- | --- | --- |
|  | | | | | | | | |  |
|  | **Data not available** | | | **Centres** | | | | | |
| **Variables** | **Number** | **Overall** | **%** | **1** | **2** | **3** | **4** | **5** | **6** |
| Age | 2695 | · |  | · | · | · | · | · | · |
| Sex | 2695 | · |  | · | · | · | · | · | · |
| ALL Lineage (B or T lineage) | 2695 | · |  | · | · | · | · | · | · |
| Bulky disease | 2695 | 64 | 2.4 | 55 | 6 | 0 | 2 | 0 | 1 |
| WBC count | 2695 | 3 | 0.1 | 0 | 2 | 0 | 0 | 0 | 1 |
| CNS disease | 2695 | 101 | 3.7 | 46 | 7 | 40 | 0 | 5 | 3 |
| Cytogenetics (BCP-ALL) | 2466 | 102 | 4.1 | 7 | 4 | 43 | 33 | 8 | 7 |
| Prednisolone Response | 2695 | 120 | 4.5 | 67 | 26 | 7 | 13 | 5 | 2 |
| End of Induction Remission status | 2557 | 17 | 0.7 | 5 | 4 | 2 | 1 | 4 | 1 |
| End of Induction MRD (BCP-ALL) | 2277 | 111 | 4.9 | 5 | 2 | 13 | 38 | 50 | 3 |
| ALL, acute lymphoblastic leukaemia; BCP-ALL, B cell-precursor acute lymphoblastic leukaemia; | | | | | | | | | |
| CNS, central nervous system; MRD, measurable residual disease; WBC, white blood cell count | | | | | | | | | |

| Table S5: Timing of relapses by ICiCLe-ALL protocol treatment phase | | | | | | | |
| --- | --- | --- | --- | --- | --- | --- | --- |
|  | | | | | | |  |
|  | **All** | **Centre 1** | **Centre 2** | **Centre 3** | **Centre 4** | **Centre 5** | **Centre 6** |
|  | n (%) | n (%) | n (%) | n (%) | n (%) | n (%) | n (%) |
| Pre maintenance | 22 (4) | 5 (2) | 0 | 9 (7) | 2 (3) | 5 (8) | 1 (3) |
| ▪ Consolidation | 5 (1) | 2 (1) | 0 | 1 (1) | 0 | 2 (3) | 0 |
| ▪ Interim maintenance | 7 (1) | 1 (0.4) | 0 | 4 (3) | 0 | 2 (3) | 0 |
| ▪ Delayed intensification | 10 (2) | 2 (1) | 0 | 4 (3) | 2 | 1 (2) | 1 (3) |
| Maintenance | 378 (60) | 173 (65) | 26 (36) | 87 (65) | 39 (63) | 34 (53) | 19 (63) |
| Post-treatment | 227 (36) | 88 (33) | 46 (64) | 37 (28) | 21 (34) | 25 (39) | 10 (33) |
| **Total relapses** | **627 (25)** | **266 (21)** | **72 (37)** | **133 (43)** | **62 (18)** | **64 (27)** | **30 (28)** |

| Table S6: Relapses within risk groups, time and site of relapse | | | | | |
| --- | --- | --- | --- | --- | --- |
|  | **All** | **SR** | **IR** | **HR** | **T ALL** |
|  | **n (%)** | **n (%)** | **n (%)** | **n (%)** | **n (%)** |
| **N** | 2475 | 593 | 626 | 1058 | 198 |
| **Total relapses** | 627 | 120 | 142 | 314 | 51 |
| **Time-point of relapse** |  |  |  |  |  |
| Very early | 243 (39) | 22 (18) | 49 (35) | 131 (42) | 41 (80) |
| Early | 269 (43) | 55 (46) | 72 (51) | 133 (42) | 9 (18) |
| Late | 115 (18) | 43 (36) | 21 (15) | 50 (16) | 1 (2) |
| **Type of relapse** |  |  |  |  |  |
| **Isolated extramedullary** | 166 (7) | 30 (5) | 33 (5) | 96 (9) | 16 (8) |
| CNS | 133 (5) | 16 (3) | 25 (4) | 82 (8) | 10 (5) |
| Testis | 29 (1) | 13 (2) | 8 (1) | 14 (1) | 3 (2) |
| CNS+Testes | 1 (0.04) | 1 (0.2) | 0 | 0 | 0 |
| Other | 3 (0.1) | 0 | 0 | 0 | 3 (2) |
| **Isolated medullary** | 298 (12) | 58 (10) | 69 (11) | 145 (14) | 26 (13) |
| **Combined** | 163 (7) | 32 (5) | 40 (6) | 73 (7) | 9 (5) |
| CNS | 114 (5) | 14 (2) | 29 (5) | 64 (6) | 7 (4) |
| Testis | 38 (2) | 16 (3) | 8 (1) | 5 (0.5) | 0 |
| CNS+Testes | 7 (0.3) | 1 (0.2) | 3 (0.5) | 2 (0.2) | 1 (0.5) |
| Other | 4 (0.2) | 1 (0.2) | 0 | 2 (0.2) | 1 (0.5) |
| **Total relapses** | 627 (25) | 120 (20) | 142 (23) | 314 (30) | 51 (26) |
| SR, Standard Risk; IR, Intermediate Risk; HR, High Risk | | | | | |
| Combined relapses bone marrow (medullary) and extramedullary sites; | | | | | |
| CNS, central nervous system | | | | | |
| Very early: relapse within 18 months from diagnosis | | | | | |
| Early: relapse >18 months from diagnosis and ≤ 6 months from end of treatment | | | | | |
| Late: relapse > 6 months from end of treatment; CNS: Central nervous system | | | | | |

| Table S7: Treatment-related deaths reported by centres at different treatment phases | | | | | | | | |
| --- | --- | --- | --- | --- | --- | --- | --- | --- |
|  | | **All** | **Centre 1** | **Centre 2** | **Centre 3** | **Centre 4** | **Centre 5** | **Centre 6** |
|  | | n (%) | n (%) | n (%) | n (%) | n (%) | n (%) | n (%) |
| **N (Induction)** | | 2695 | 1393 | 209 | 342 | 364 | 274 | 113 |
| Induction death | | 127 (5) | 64 (5) | 7 (3) | 24 (7) | 19 (15) | 10 (4) | 3 (3) |
| **N (Post-Induction)** | | 2475 | 1286 | 193 | 307 | 341 | 241 | 107 |
| Post Induction death | | 169 (7) | 73 (6) | 6 (3) | 24 (8) | 42 (12) | 13 (5) | 11 (10) |
| Pre maintenance | | 55 (2) | 20 (2) | 1 (0·5) | 3 (1) | 20 (6) | 5 (2) | 6 (6) |
| ▪ Consolidation | | 27 (1) | 7 (1) | 1 (0·5) | 1 | 11 (3) | 3 (3) | 4 (4) |
| ▪ Interim maintenance | | 14 (1) | 6 (1) | 0 | 2 (1) | 2 (1) | 2 (1) | 2 (2) |
| ▪ Delayed intensification | | 14 (1) | 7 (1) | 0 | 0 | 7 (2) | 0 | 0 |
| Maintenance | | 103 (4) | 50 (4) | 3 (2) | 20 (7) | 21 (6) | 4 (2) | 5 (5) |
| Post-treatment | | 11 (0·4) | 3 (0·2) | 2 (1) | 1 (0·3) | 1 (0·3) | 4 (1·7) | 0 |
| **Total death** | | **296 (11)** | **137 (10)** | **13 (6)** | **48 (14)** | **61 (17)** | **23 (8)** | **14 (12)** |
|  |  | |  |  |  |  |  |  |

# Table S8A-B

| Table S8A: EFS of patients, by treatment centre | | | | |  |  |
| --- | --- | --- | --- | --- | --- | --- |
|  |  |  |  |  |  |  |
| Centres | 4 year EFS (95% CI) | p value* |  |  |  |  |
| 1 | 67.1 (64.5 - 69.6) | **<0.0001** |  |  |  |  |
| 2 | 54.6 (46.8 - 61.6) |  |  |  |  |  |
| 3 | 42.7 (36.6 - 48.6) |  |  |  |  |  |
| 4 | 65.7 (60.0 - 70.8) |  |  |  |  |  |
| 5 | 63.7 (56.8 - 69.7) |  |  |  |  |  |
| 6 | 54.6 (43.2 - 64.7) |  |  |  |  |  |
| 95% CI, 95% confidence interval; *Log-rank test | | |  |  |  |  |
|  |  |  |  |  |  |  |
| Table S8B: Pairwise EFS comparisons between centres (log-rank test) | | | | | | |
| Centre | 1 | 2 | 3 | 4 | 5 | 6 |
| 1 | · | **0.002** | **<0.0001** | 0.6 | 0.1 | **0.02** |
| 2 | · | · | **0.003** | **0.04** | 0.2 | 0.8 |
| 3 | · | · | · | **<0.0001** | **<0.0001** | 0.05 |
| 4 | · | · | · | · | 0.4 | 0.08 |
| 5 | · | · | · | · | · | 0.3 |
| 6 | · | · | · | · | · | · |

# Table S9: Data for Figures

|  |  |  |  |  |  |  |  |  |  |  |  |  |
| --- | --- | --- | --- | --- | --- | --- | --- | --- | --- | --- | --- | --- |
|  | |  |  |  |  |  |  |  |  |  |  |  |
| **Data for Figure 3A** | |  |  |  |  |  |  |  |  |  |  |  |
| **Centres** | **Months** | **0** | **6** | **12** | **18** | **24** | **30** | **36** | **42** | **48** | **54** | **60** |
| **1** | Number at risk | 1393 | 1284 | 1212 | 1136 | 1057 | 970 | 836 | 698 | 595 | 499 | 388 |
|  | Number of deaths | 0 | 82 | 103 | 115 | 123 | 129 | 133 | 134 | 134 | 134 | 134 |
| **2** | Number at risk | 209 | 195 | 187 | 177 | 169 | 162 | 135 | 119 | 116 | 112 | 107 |
|  | Number of deaths | 0 | 8 | 8 | 10 | 11 | 11 | 11 | 11 | 11 | 11 | 11 |
| **3** | Number at risk | 342 | 299 | 270 | 229 | 197 | 179 | 164 | 149 | 134 | 128 | 121 |
|  | Number of deaths | 0 | 27 | 31 | 35 | 41 | 44 | 45 | 46 | 47 | 47 | 47 |
| **4** | Number at risk | 364 | 322 | 303 | 276 | 256 | 229 | 212 | 199 | 187 | 170 | 141 |
|  | Number of deaths | 0 | 33 | 45 | 47 | 53 | 59 | 59 | 59 | 60 | 60 | 60 |
| **5** | Number at risk | 274 | 249 | 233 | 221 | 211 | 200 | 190 | 180 | 176 | 173 | 147 |
|  | Number of deaths | 0 | 15 | 17 | 17 | 18 | 19 | 19 | 19 | 19 | 19 | 19 |
| **6** | Number at risk | 113 | 99 | 88 | 78 | 73 | 69 | 62 | 51 | 44 | 34 | 27 |
|  | Number of deaths | 0 | 6 | 10 | 10 | 12 | 13 | 13 | 14 | 14 | 14 | 14 |
| **Data for Figure 3B** | |  |  |  |  |  |  |  |  |  |  |  |
| **Centres** | **Months** | **0** | **6** | **12** | **18** | **24** | **30** | **36** | **42** | **48** | **54** | **60** |
| **1** | Number at risk | 1393 | 1284 | 1212 | 1136 | 1057 | 970 | 836 | 698 | 595 | 499 | 388 |
|  | Number of relapses | 0 | 5 | 38 | 88 | 142 | 183 | 226 | 254 | 262 | 264 | 266 |
| **2** | Number at risk | 209 | 195 | 187 | 177 | 169 | 162 | 135 | 119 | 116 | 112 | 107 |
|  | Number of relapses | 0 | 0 | 7 | 16 | 22 | 27 | 51 | 63 | 66 | 68 | 68 |
| **3** | Number at risk | 342 | 299 | 270 | 229 | 197 | 179 | 164 | 149 | 134 | 128 | 121 |
|  | Number of relapses | 0 | 9 | 30 | 64 | 86 | 98 | 110 | 121 | 127 | 128 | 129 |
| **4** | Number at risk | 364 | 322 | 303 | 276 | 256 | 229 | 212 | 199 | 187 | 170 | 141 |
|  | Number of relapses | 0 | 2 | 9 | 30 | 35 | 45 | 53 | 59 | 61 | 61 | 61 |
| **5** | Number at risk | 274 | 249 | 233 | 221 | 211 | 200 | 190 | 180 | 176 | 173 | 147 |
|  | Number of relapses | 0 | 5 | 16 | 27 | 33 | 42 | 49 | 58 | 59 | 60 | 62 |
| **6** | Number at risk | 113 | 99 | 88 | 78 | 73 | 69 | 62 | 51 | 44 | 34 | 27 |
|  | Number of relapses | 0 | 1 | 7 | 17 | 20 | 22 | 25 | 27 | 29 | 30 | 30 |

# Table S10: Cumulative incidence of relapse in provisional non-high risk B cell-precursor acute lymphoblastic leukaemia patients (BCP-ALL), stratified by levels of bone marrow minimal residual disease at end of induction (EoI-MRD).

| BCP-ALL  Provisional Risk | EoI-MRD | Cum Inc Relapse % (95% CI) | ***p**** |
| --- | --- | --- | --- |
| Standard & Intermediate | High | 33 (27-38) | **0·003** |
|  | Low | 22 (20-25) |  |
| Standard | High | 27 (20-34) | 0·17 |
|  | Low | 20 (17-24) |  |
| Intermediate | High | 41 (32-50) | **0·002** |
|  | Low | 25 (21-28) |  |
| Cum Inc Relapse, cumulative incidence of relapse | | | |
| 95% CI, 95% confidence interval; | | | |
| EoI MRD; high (≥ 0·01%), low (<0·01%) | | | |
| *Gray test, with treatment-related death as competing risk | | | |

# Table S11: Outcomes of previous treatment protocols for newly diagnosed paediatric acute lymphoblastic leukaemia in India

|  |  |  |  |  |  |  |  |
| --- | --- | --- | --- | --- | --- | --- | --- |
| Treatment Centre (City) | Mumbai | Delhi | Chennai | Vellore | Chennai | Chandigarh | Chandigarh |
| Treatment protocol | MCP841 | MCP841 | MCP841 | BFM 76/79 | BFM 95 | UKALL 2003 | mod UKALL 2003 |
| Patient age | <25 years | <25 years | <25 years | <15 years | <30 years | <14 years | <14 years |
| Study timeline | 1990 - 1997 | 1990 - 1997 | 1990 - 1997 | 1985 - 2003 | 2005 - 2011 | 2007 - 2009 | 2010 - 2011 |
| Patients | 652 | 228 | 168 | 298 | 238 | 190 | 184 |
| Treatment related death | 66 (10%) | 53 (23%) | 33 (20%) | 11 (4%) | · | 70 (36%) | 38 (21%) |
| • Induction death | · | · | · | 6 (2%) | 8 (3%) | 20 (11%) | 16 (9%) |
| • Post-induction death | · | · | · | 5 (2%) | · | 50 (26%) | 22 (12%) |
| Relapse | 192 (30%) | 65 (29%) | 65 (39%) | 83 (28%) | 74 (31%) | 29 (15%) | 27 (15%) |
| Event free survival (EFS) | 60% | 41% | 43% | 56% | 63% | 51% | 66% |
| • EFS time | 4 years | 4 years | 4 years | 5 years | 3 years | 3 years | 3 years |
| Abandoned/Loss to follow-up | · | · | · | 30 (10%) | 8 (3%) | 4 (2%) | 4 (2%) |
| Year of publication | 2004 | 2004 | 2004 | 2008 | 2015 | 2017 | 2017 |
| Reference | 1 | 1 | 1 | 2 | 3 | 4 | 4 |
| Reference 1: Eur J Cancer. 2005;41:1570-1583; Reference 2: Pediatr Blood Cancer. 2008;51:621-626 | | | | | |  |  |
| Reference 3: Indian J Med Paediatr Oncol. 2015;36:261-264; Reference 4: Pediatr Blood Cancer. 2017;64(4) (mod UKALL, modified UKALL) | | | | | | | |

# Supplementary Figure Legends

**Supplementary Figure S1**: Cumulative incidence of induction deaths in ALL risk groups.

Risk groups include provisional standard risk (SR), provisional intermediate risk (IR) and high risk (HR) B cell-precursor acute lymphoblastic leukaemia patients and patients with T-ALL. Values represent 3-year estimates with 95% confidence intervals, compared using the Gray test

**Supplementary Figure S2:** Cumulative incidence of relapse at 36 months and 48 months (values with 95% confidence intervals) estimated from time of diagnosis in BCP-ALL and T-ALL patients treated on the ICiCLe-ALL multicentre observational study (Gray test for comparison of estimates, treatment-related death as competing risk)

**Supplementary Figure S3**: Overall survival post-induction in Standard (593 patients), Intermediate (626 patients) and High Risk (1058 patients) B cell-precursor ALL patients

Kaplan-Meier curves; 3-year estimate (95% confidence interval) from final risk stratification post-induction; log-rank test for comparison of survival estimates

# Figure S1

# Figure S2

**
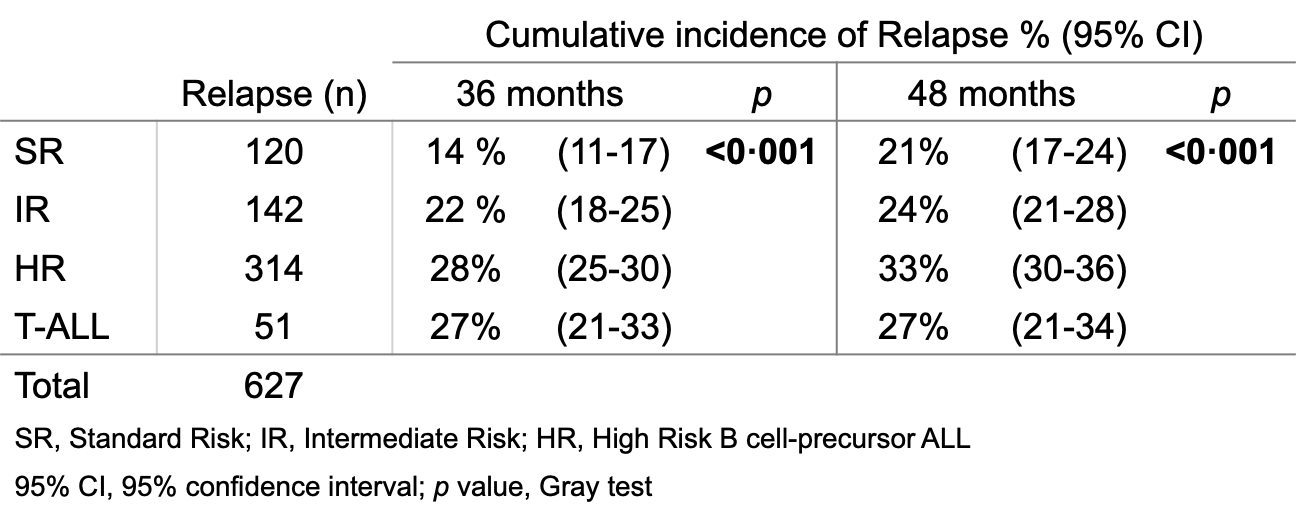
**

# Figure S3
